# The Neural Basis of Birdsong

**DOI:** 10.1371/journal.pbio.0030164

**Published:** 2005-05-17

**Authors:** Fernando Nottebohm

## Abstract

Songbirds represent an excellent model system for understanding the neural mechanisms underlying learning.

There is a tradition in biology of using specific animal models to study generalizable basic properties of a system. For example, the giant axon of squid was used for the pioneering work on nerve transmission; the fruit fly (Drosophila) has played a key role in researchers discovering the role of homeobox genes in embryogenesis; the sea slug (Aplysia) is used to study the molecular biology of learning; and the round worm (Caenorhabditis elegans) is used to study programmed cell death. Basic insights gained from these four systems apply widely to other multicellular animals. Here, I will review basic discoveries made by studying birdsong that have helped answer more general questions in vertebrate neuroscience.

## Vocal Learning and the “Song System”

Oscine songbirds (e.g., zebra finches, canaries, and white-crowned sparrows) learn their song by imitating those of older members of their own species [1,2]. This is done by modifying vocal output until the auditory feedback it generates matches a memorized model [3]. In some birds vocal learning gives rise to easily discernible song dialects, which then act as local cultural traditions [4]. In most songbirds mastery of a song model takes many weeks. Song learning starts with a stage that has been likened to human infant babbling called “subsong,” during which highly variable, low-amplitude sounds are produced in a non-communicatory context, often while the juvenile seems to doze. The sounds of subsong provide the raw material from which imitations emerge. As these imitations become recognizable, they are referred to as “plastic song.” As the imitations are perfected, song becomes less and less variable. The stable song typical of adults is in place by the time the sexually mature bird is ready to start to defend a territory and woo a mate. Intriguingly, in birds as in human infants, the path of vocal change that culminates with imitation of a model can be very idiosyncratic, as if this were an exercise in problem solving for which there is no single solution [5].

**Figure 1:**
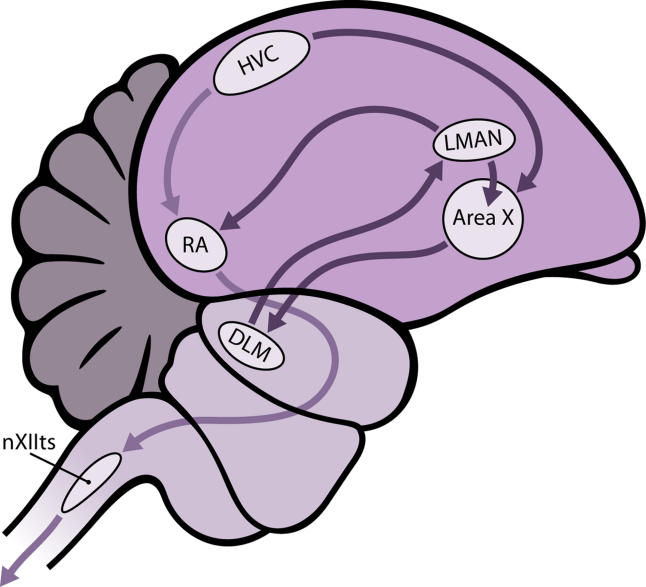
The Song System of Songbirds Nucleus HVC feeds information into two pathways that ultimately lead to the neurons in the tracheosyringeal half of the hypoglossal nucleus (nXIIts) that project to vocal muscles. HVC projects to nucleus RA directly (PDP), and indirectly via Area X, the dorsolateral anterior thalamic nucleus (DLM), and LMAN (AFP) in a manner that shares similarities with the mammalian pathway cortex→basal ganglia→thalamus→cortex.

The acquisition and production of learned song is made possible by a group of discrete brain nuclei and their connecting pathways, referred to as the “song system” [6,7], which has similarities in the three groups of birds—songbirds, parrots, and hummingbirds—that evolved learned song [8,9]. This system, described in considerable detail in oscine songbirds, has two main branches: the posterior descending pathway (PDP), necessary for both the acquisition and production of learned song, and the anterior forebrain pathway (AFP), necessary for acquisition only (see Figure 1). The high vocal center (HVC) is at the starting point of both these pathways, but the HVC cells that project to the PDP and AFP differ. In mammalian terms the PDP is homologous to a motor pathway that starts in the cerebral cortex and descends through the brain stem [6], while the AFP is homologous to a cortical pathway through the basal ganglia and thalamus [7,10,11].

Several of the telencephalic nuclei that participate in the production and acquisition of learned song are small in nestlings, before the onset of song development, and their volume, cell number, cell size, and connections grow during the subsequent weeks or months. As a result of these changes, many of the components of the circuits for the acquisition and production of learned song are formed and connected during the very period when song first develops (reviewed in [12]). Another peculiarity of this system is that the right and left sides of the brain can operate, to some extent, independently, each responsible for a different array of sounds. In birds such as the canary, the chaffinch, and the white-crowned sparrow a majority of the sounds of song are produced by the left syringeal half, under the control of left, uncrossed pathways. This phenomenon has been referred to as “left hypoglossal” or “left hemispheric” dominance [13].

## Adult Variation, Neurogenesis, and Neuronal Replacement

The song system of birds is sexually dimorphic: it is better developed in males, which usually sing more and produce a more complex repertoire than females. For example, the nucleus HVC of canaries is three times larger in males than in females; in zebra finches it is eight times larger [14]. In seasonal singers such as the canary and song sparrow, song system nuclei such as HVC are significantly larger in the spring than in late summer, after breeding stops [15,16]. Cells in many of the song control nuclei are androgen and estrogen sensitive [17]. The nucleus HVC of adult female canaries treated with physiological doses of testosterone doubles in volume, and these birds start to sing in a male-like manner [18]. Initially such changes were thought to result solely from growth of dendritic trees and synapse formation [19], but subsequently it was found that new neurons were added, too. These new neurons, as in embryos, are born in the wall of the forebrain's lateral ventricle [20,21]. Interestingly, the addition of new neurons to nucleus HVC occurred also in male and female adult canaries that had received no hormonal treatment [22].

Earlier claims of neurogenesis in the adult mammalian brain [23,24] had met resistance [25]. Nucleus HVC yielded the first unambiguous example of adult neurogenesis, in that individual cells labeled with a cell birth marker provided neurophysiological recordings that were unmistakably neuronal [26]. We now know that the recruitment of new HVC neurons is part of a process of constant replacement [27,28]. This replacement is particularly active in canaries during seasonal changes in the song repertoire [29]. Male canaries develop a new song repertoire each year. New neurons are constantly added, as well, to many regions of the adult avian telencephalon, where they are probably involved in a variety of brain functions. There is no evidence of neuronal addition to other parts of the adult songbird brain [22]. We now know that adult neurogenesis and neuronal replacement are probably common to all vertebrates [30]. The song system of birds helped change the way in which we think of brain circuits and their potential for rejuvenation and repair. Just as important, the discovery of neuronal replacement has raised basic questions about the brain variables that set limits to learning [31].

## Neurophysiology Offers Insights on the Mechanisms for Vocal Learning

From early on, the song system drew the attention of neurophysiologists. It was known that lesions of HVC and the robust nucleus of the arcopallium (RA) affected the organization of song differently, the former being more devastating than the latter [6]. Likewise, stimulation of HVC during song interrupted and reset the song program, something that did not happen if the stimulating electrode was in RA [32]. This hierarchical relation between HVC and RA was confirmed by recording from HVC and RA while the bird sang [33], but the manner in which the sounds of song were represented in HVC remained unclear. This issue was resolved by recording from individual HVC neurons that projected to nucleus RA. These neurons, it was shown, fired very sparsely and at narrowly defined times, each neuron firing always during the same six-millisecond window while the bird produced its single learned song [34]. The inference that these neurons—and the PDP of which they are part—carried the learned pattern of song seems inescapable. Since these are the very HVC neurons that are replaced when birds modify their song [31], it follows that the replacement cells learn their score.

But what, then, is the role of the AFP in song learning? It was known that the AFP was necessary for the acquisition but not for the production of learned song [35]. It was known, too, that the variable song typical of juvenile songbirds became very stereotyped after bilateral lesions of the lateral magnocellular nucleus of the nidopallium (LMAN) (part of the AFP), from which it was inferred that LMAN played a crucial role in fostering circuit plasticity necessary for learning [36]. But the mechanism for this effect remained unknown. Two recent independent studies now show how this effect comes about [37,38]. In this issue of *PLoS Biology*, Ölveczky and colleagues show that the LMAN neurons that project to RA fire in a quasi-random pattern when variable song is produced in juvenile birds. Thus, while the HVC→RA projection carries the learned song, the LMAN→RA projection carries the jitter that induces the variability in motor output necessary for the imitation of a model. This jitter, presumably, is imposed on the firing of the same RA neurons that receive the more orderly output from HVC. When the LMAN neurons are silent (or absent), the HVC→RA pathway produces a stereotyped pattern; when the LMAN→RA neurons are firing, song is more variable. This is a most elegant breakthrough. However, it is not clear exactly how this pathway functions in song learning. One possibility is that birds trying to imitate a model succeed by retaining, from the variability generated, those patterns that more closely approximate the model and discard the rest, thus, over a period of time, achieving a perfect imitation. A second possibility is that the variable mismatch between a model and the attempted imitation drives output modification, so that patterns that had not occurred before now first appear. Both mechanisms would depend on auditory feedback. It is the first time we are so close to a mechanism for vocal learning.

## Open Questions

The stage is set for many more insights. An unsolved question is the extent to which the very pathways that produce learned song may also partake in the perception of song. On a different front, why is it that HVC→RA neurons are periodically replaced? It was thought that changes in dendritic configuration, dendritic spines, and synaptic number and efficiency provided all the plasticity needed to change circuit configuration and explain how new information was acquired and remembered. But if so, why replace whole neurons? Is it possible that dendritic and synaptic changes underlying learning are less easy to achieve in older neurons? If so, are older neurons replaced to reinstate a level of plasticity necessary for learning? Or might it be that in some cases the whole neuron, rather than the synapse, is the unit of learning? In this latter scenario, changes associated with learning would be committed as permanent gene expression changes akin to those characterizing cellular differentiation. Such a change would be a very stable way to encode learning, but it would have a major drawback: the more learning that occurred, the fewer neuronal pupils would remain. Thus, we are left to wonder whether neuronal replacement takes place to make up for the lost plasticity of aging neurons, or whether it takes place as part of a normal recycling of memory space and of the memories it holds.

During the early 1970s, before the song system was discovered, it was widely believed that the learning of any one skill had a wide representation in the vertebrate brain [39]. The discovery of discrete brain regions devoted to song learning and execution in the bird brain helped change that view. It was also widely believed that the brains of male and female vertebrates were virtually identical, with small allowances for the levels of circulating hormones. The song system changed that, too. And it was widely believed that the anatomy of adult brains was set, but we now know that the volume of brain structures can change seasonally and in response to blood hormone levels. Most importantly, it was widely held that though a straggling few neurons might still be added after birth to late-developing parts of the brain, brain cells, once lost, could not be replaced. Again, work on the song system changed the prevailing view. It may well be that our best understanding of how complex skills are acquired and how broken circuits can be fixed will come not from humans, or other primates, but from the way birds learn their song.

## Abbreviations

AFP: anterior forebrain pathway
HVC: high vocal center
LMAN: lateral magnocellular nucleus of the nidopallium
PDP: posterior descending pathway
RA: robust nucleus of the arcopallium
